# The Role of Irisin, Insulin and Leptin in Maternal and Fetal Interaction

**DOI:** 10.4274/jcrpe.0096

**Published:** 2018-11-29

**Authors:** Deniz Ökdemir, Nihal Hatipoğlu, Selim Kurtoğlu, Ülkü Gül Siraz, Himmet Haluk Akar, Sabahattin Muhtaroğlu, Mehmet Serdar Kütük

**Affiliations:** 1 Fırat University Faculty of Medicine, Department of Pediatric Endocrinology, Elazığ, Turkey; 2 Erciyes University Faculty of Medicine, Department of Pediatric Endocrinology, Kayseri, Turkey; 3 Çanakkale Onsekiz Mart University Faculty of Medicine, Department of Pediatric Allergy, Çanakkale, Turkey; 4 Erciyes University Faculty of Medicine, Department of Biochemistry, Kayseri, Turkey; 5 Erciyes University Faculty of Medicine, Department of Perinatology, Kayseri, Turkey

**Keywords:** Irisin, insulin, leptin, fetal growth, maternal weight gain

## Abstract

**Objective:**

Insulin is an important hormone for intrauterine growth. Irisin is an effective myokine in the regulation of physiological insulin resistance in pregnancy. Leptin and insulin are associated with fetal growth and fetal adiposity. In this study, we aimed to investigate the relationships between irisin, insulin and leptin levels and maternal weight gain, as well as anthropometric measurements in the newborn.

**Methods:**

Eighty-four mothers and newborns were included in the study. Irisin, leptin and insulin levels were measured in the mothers and in cord blood. Anthropometric measurements in the newborn, maternal weight at the beginning of the pregnancy and at delivery were recorded.

**Results:**

Birth weight were classified as small for gestational age (SGA), appropriate for gestational age (AGA) and large for gestational age (LGA). There was no difference in irisin levels among the groups. Leptin and insulin levels were found to change significantly according to birth weight (p=0.013, and p=0.012, respectively). There was a negative correlation between the anthropometric measurements of the AGA newborns and irisin levels. This correlation was not observed in SGA and LGA babies. Leptin levels were associated with fetal adiposity.

**Conclusion:**

While irisin levels are not affected by weight gain during pregnancy nor by birth weight, they show a relationship with anthropometric measurements in AGA infants. These results may lead to the understanding of metabolic disorders that will occur in later life.

**What is already known on this topic?**

Irisin regulates physiological insulin resistance in pregnancy. The effects of irisin on fetal growth have not yet been completely elucidated. It is known that obesity, type 2 diabetes and the metabolic syndrome, insulin resistance and consequently increased risks of cardiovascular disease can be programmed in the intrauterine period.

**What this study adds?**

Cord blood levels of irisin, insulin, and leptin were associated with fetal growth. These findings may help to identify the risks that may arise in the later stages of life due to intrauterine growth problems.

## Introduction

Boström et al (1) first discovered irisin, a myokine, in 2012 and they reported that irisin stimulates the transition of white fat tissue to brown fat tissue. Increased levels of irisin by exercise and exposure to cold leads to the production of fibronectin-type 3 domain-containing protein 5 (FNDC5), a membrane protein (1,2,3). Obese people with higher levels of basal irisin were shown to lose more weight with dieting and increased levels of irisin with early intervention in obesity have been reported to reduce insulin secretion and blood sugar level (4). The post acute exercise level was temporarily increased, while the level of prolonged exercise decreased and was higher in prediabetics than in controls. Moreover, it has been shown that the level of irisin can be heritable and that there is an irisin-related positive correlation between mother and son (5).

There are also various reports on associations of irisin with chronic disease processes such as type-2 diabetes, metabolic syndrome, cardiovascular diseases, osteoporosis, chronic kidney disease, proliferation of cancer cells and nonalcoholic fatty liver (6). It has been reported that irisin has an effect in increasing energy expenditure, weight loss, glucose and insulin resistance and that it exerts these effects by stimulating the transformation of white adipose tissue to brown-like adipose tissue (1,7).

Brown fat tissue is important in thermogenesis and energy metabolism. The amount of brown fat tissue is high in the neonatal period (8). Intrauterine growth restriction (IUGR) creates a predisposition to fetal fat tissue changes in fetal growth disorders leading to macrosomia, permanent hormonal changes and obesity and insulin resistance in future years (9,10). IUGR also causes a disproportion in fat mass compared to lean mass, and retardation in growth and skeletal muscle development (11,12). IUGR infants have high insulin sensitivity at birth and are predisposed to insulin resistance after rapid growth in the postnatal period (13). In addition, large for gestational age (LGA) infants are predisposed to obesity and cardiovascular disease later in life as they develop a high fat ratio and reduced insulin sensitivity (14).

Irisin has an important role in controlling maternal and fetal glucose hemostasis. It has been found that the growth of newborns with low irisin levels in their cord blood is delayed and that the proportion of their brown fat tissue is low. Thus, irisin may play an important role in the regulation of maternal-fetal glucose hemostasis (15).

The pregnancy period is a period of increased oxidative stress and it has been shown that irisin reduces oxidative stress (16,17,18). Accordingly, the level of irisin in pregnant women is higher than that of non-pregnant women (19). It is thought that irisin also contributes to the physiological insulin resistance found in pregnancy (19).

Babies of obese mothers have a higher incidence of LGA birth and are more at risk for obesity in the future due to intrauterine programming (14). Maternal leptin levels and newborn fat mass percentiles are interrelated (20). Maternal leptin level increases during pregnancy, as a consequence of placental production, and this increase is associated with fat mass gain (21,22). Leptin also has an effect on fetal adiposity (20).

The purpose of this study was to investigate the relationships between maternal weight at the beginning of pregnancy and weight gain during pregnancy, birth weight, anthropometric measurements; and the effects of these on irisin, leptin and insulin levels.

## Methods

This study was conducted on mothers who delivered in the Perinatology Department of the Erciyes University Medical Faculty and their offspring. Signed consent was obtained from all women who volunteered to participate in the study. The study was approved by the University’s Ethics Committee (date of approval: 04/04/2014, approval number: 234).

Mothers with diabetes, hypothyroidism, chronic kidney disease, epilepsy, chronic liver disease, hypertension, chronic drug use, asthma, smoking and those with placental and fetal problems were excluded. Premature births (<38 weeks) were excluded from the study. The mother’s weight before and after pregnancy was obtained from the hospital files. A proportion of the births were elective cesarean sections. Blood samples were taken simultaneously from maternal venous fasting blood (8 hours fasting) and postnatal fetal umbilical artery under sterile conditions. These blood samples were centrifuged at 5,000 rpm for 5 minutes and stored at -80 °C, and all the samples were analyzed together. Anthropometric measurements were taken on all infants after delivery by the same physician using the same scale.

Ponderal index (PI) was calculated using the [birth weight (g) / birth height (cm^3^) x 100] formula in all infants. Those with a PI <2.32 were classified as of low birth weight (SGA), those between 2.32 and 2.85 were classified as of normal birth weight (AGA), and those with >2.85 as overweight birth weight (LGA) (23).

Irisin levels were measured in serum using a commercial human enzyme-linked immunosorbent assay (ELISA) kit (BioVendor, Heidelberg, Germany). The measurement range of this assay was between 0.01 and 100 µg/mL.

Maternal and infant serum leptin levels were measured using a human ELISA kit (DIAsource ImmunoAssays S.A., Rue du Bosquet, 2, B-1348 Louvain-la-Neuve, Belgium). Serum insulin levels were measured using the Dia Metra kit for immunoenzymatic determination.

### Statistical Analysis

Statistical analysis of the data was performed with the IBM SPSS 22.0 program (IBM Inc., Chicago, Ill., USA). The normality distributions of the groups were determined according to the Kolmogorov-Smirnov test because the number of cases was small. Since the distribution between groups was not normal, the data were given as median and range. Mann-Whitney U test was used for nonparametric tests in comparison of variables including continuous data. The p value was expected to be less than 0.05 for significance. Kruskal Wallis variance analysis was applied as a post-hoc test between the groups. Bonferroni correction was applied for post hoc analyzes and a p value of <0.0125 was taken as significant. Spearman correlation analysis was used to evaluate the relationship between the two continuous variables.

## Results

Eighty four mothers with a mean age of 29.8±5.2 years and their newborns were enrolled in the study. The mean duration of pregnancy was 38.7±0.9 weeks. 53.6% of the births were cesarean delivery. At the beginning of the pregnancy, 32.1% of the mothers were overweight, while 15.5% were obese and 27.4% of them gained too much weight during pregnancy (>15 kg). Forty six (55%) of the newborns were male and 38 (45%) were female.

9.5% of the infants were classified as SGA, 73.8% as AGA and 16.7% as LGA. When the anthropometric and hormonal values of the mother and the baby were compared among these three groups, significant differences were found between all anthropometric parameters except for the birth length of the infants (Table 1). Hormonal levels for leptin and insulin differed among the infants in the 3 groups, but no differences were found in other parameters (Table 1).

Birth weight, PI values, head, neck, middle arm circumference and skin fold thicknesses of the infants were significantly higher in the infants who were the offspring of mothers who were overweight when the mothers were divided into two groups according to weight they gained in pregnancy (Table 2). However, maternal weight gain did not cause a difference in hormone levels in either mothers or infants (Table 2).

When the relationship between maternal and infant anthropometric measurements and hormonal changes were analyzed, while there was no relationship between pre-pregnancy body mass index (BMI) values and anthropometric values of the infants, a statistically significant positive correlation was found between these values and the PI, abdomen and arm circumference of the infants and post-pregnancy BMI score and between PI and the infants’ head, chest, abdomen, left mid arm circumference and skinfold thicknesses (triceps and biceps) (Table 3).

**Figure 1:**
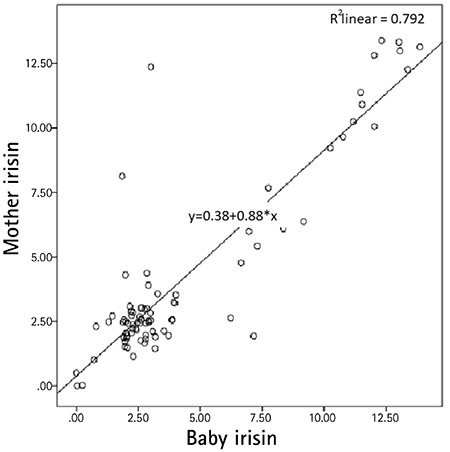
Correlation analysis between mother and baby irisin levels

When assessed in terms of the relationships between mother-infant hormone changes and anthropometric measurements, there was a strong positive correlation between maternal-infant irisin levels and a moderately strong positive correlation between mother-infant insulin and leptin levels (Figure 1). There was a weak negative correlation between maternal irisin levels and infant chest, neck, and left middle arm circumference values. There was no relationship between maternal insulin and leptin levels and anthropometric measurements of the infants. There was a positive correlation between the leptin levels of the infants and all anthropometric measurements and between insulin levels and PI, neck circumference and skinfold thickness. Only the neck circumference showed a weak negative correlation with irisin levels of the infants (Table 4).

When the mothers were classified according to weight gain in pregnancy and anthropometric measurements in the infants, hormonal interactions are observed; there was a negative correlation between the irisin levels of the mothers of normal weight-gain during pregnancy and the baby’s chest, neck, arm circumference, triceps thickness and a positive correlation between maternal insulin levels and the head circumference of the infants.

**Figure 2:**
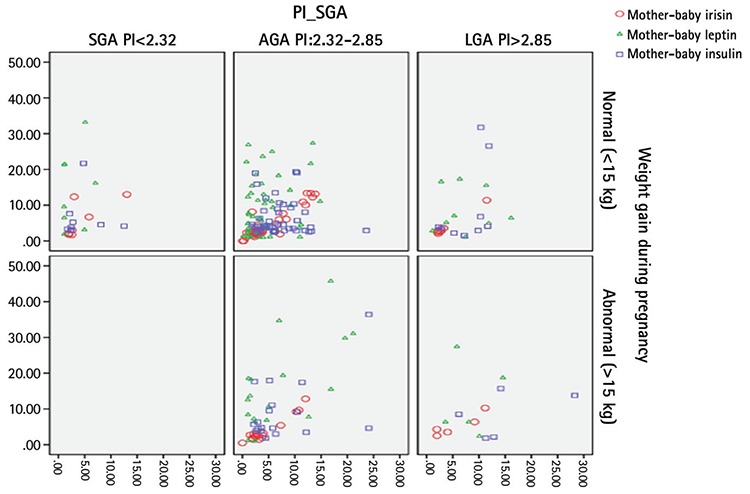
Correlation analysis of hormonal changes in the classification according to the weight gain of the mother during pregnancy and the birth weight of the baby SGA: low birth weight, AGA: normal birth weight

There was a negative relationship between irisin levels of the infant and neck circumference, a weak positive relationship between insulin levels and middle arm circumference; leptin levels were correlated with all anthropometric values showing a moderate to strong positive correlation. However, these relationships, including the mothers’ hormone levels and the infants’ anthropometric measurements, disappeared in the infants of mothers who gained excess weight during pregnancy (Figure 2).

When the babies were classified according to PI and the relationship between hormones and anthropometric measurements was analyzed, a more significant relationship was observed in the analysis of babies born with normal weight, a finding similar to the analysis according to maternal weight gain in pregnancy. Interestingly, in SGA infants, a positive correlation was observed between maternal irisin level and middle arm circumference while in the normal weight group this correlation, as well as correlations between all anthropometric measurements and skinfolds, were negative. Again, the relationship between baby irisin levels and some of the baby anthropometric measurements was negative. In LGA infants positive correlations were found between maternal leptin and infant PI, and also between maternal insulin levels and infant neck circumference. Maternal and infant hormone levels correlated with each other in all three groups (Figure 2).

## Discussion

It is known that there is a relationship between weight gain of the mother during pregnany and the birth weight of the baby (24). In this study, the interaction of maternal and infant irisin, leptin and insulin levels and factors affecting these values were investigated. It has been shown that normal weight gain in pregnancy and a AGA are influenced by these three hormones, but the effect of these hormones on the infant’s development and fat distribution is not so clear when the mother is overweight or the baby deviates from the norm to SGA or LGA.

Irisin is a hormone associated with brown fat tissue and is affected by nutrition, exercise and heat. Elevated fasting plasma glucose and increased levels of irisin have been suggested to play a protective role for insulin resistance (25,26). Physical exercise leads to an increase in peroxisome proliferator-activated receptor-g coactivator-1 alpha (a transcriptional coactivator) in the muscle tissue as well as increases in FNDC5 and irisin secretion (5).

Increase in irisin increases glucose transport. In addition, by increasing the brown fat tissue and by thermogenesis, glycolysis increases the consumption of glucose and lipids as energy by increasing oxidative phosphorylation (27). Zhang et al (28) found a significant reduction in irisin levels in type 2 diabetic patients.

Infants born as IUGR or LGA are known to develop obesity and subsequent susceptibility to insulin resistance and metabolic syndrome in later life (10,11). However, there may be signs predictive of obesity in later life in the intrauterine period (15). The mechanism of physiological insulin resistance in pregnancy is not fully understood (29). Parallel to the growth of the fetoplacental unit during the gestational period, insulin sensitivity decreases, and progressive weight gain continues (30). Maternal insulin resistance is an important mechanism for fetal growth (30). However, further increases in insulin resistance in the gestational period may cause abnormal fetal growth, fetal macrosomia and IUGR (31,32). Irisin plays a role in the regulation of this physiological insulin resistance in pregnancy and thus in intrauterine growth (33). Leptin is also effective in the development of fetal adiposity (20).

It is known that irisin has an effect on the normal growth of the baby in the fetal period. However, there are conflicting results among the studies in this area. In a study by Briana et al (33), no association was found between maternal irisin levels, BMI and insulin levels in SGA, LGA, and AGA newborns. In contrast Keleş and Turan (34) reported lower irisin levels in SGA infants than those in AGA infants.

In our study, we found no differences in relationships between birth weight and maternal and baby irisin levels in SGA, AGA and LGA infants. Similarly, irisin levels were similar when evaluated according to weight gain during pregnancy. A strong linear correlation was found between maternal and infant irisin levels consistent with previous studies. There was a weak negative relationship between maternal irisin levels and the infant’s chest, neck and arm circumference. This relationship disappeared in mothers who gained excessive weight during pregnancy. While the negative relationship between maternal and infant irisin levels and anthropometric measurements became more obvious in normal weight mothers and infants, interestingly, strong positive correlations were found between maternal irisin and baby arm circumference values in SGA infants, and PI values in LGA infants. With these associations, it can be concluded that irisin can control the baby’s adiposity in normal pregnancy weight gain in women of normal weight, but that this control is lost in infants who deviate from the norm (such as SGA and LGA) and this may have pathological effects on fat content and fat distribution.

Leptin is a hormone that correlates with the amount of white fat tissue. Castro et al (35) found no association between maternal leptin levels and fetal adiposity. In our study, although maternal leptin levels correlated with leptin and insulin levels in infants, maternal leptin did not have any effect on the fat distribution parameters of the baby. However, leptin levels differed according to PI, mostly in LGA-born infants. In addition, leptin was found to be the only hormone correlated with all anthropometric parameters of the infant. This correlation was not found to be related to any measurement parameters of either the maternal leptin levels in mothers who gained more weight nor to leptin levels in the infants; while it continued to correlate in the mothers with normal weight gain and AGA infants. Leptin levels were found to correlate with the baby’s arm circumference in SGA infants and PI in the LGA babies. This may be related to the development of a kind of resistance which occurs with disappearance of leptin due either to the pathologic weight gain of the mother or to SGA or high birth weight.

The positive effect of insulin on growth in the fetal period is well documented (10,13). Consistent with this, in our study, insulin levels of LGA-born babies were significantly higher. However, in general, we did not find a significant association between insulin levels and irisin levels. This result is consistent with the existing information in the literature (33).

### Study Limitations

An important limitation of the study was the low numbers of SGA and LGA cases. This was due to the exclusion of infants with chronic illness from the study.

## Conclusion

In conclusion, a strong correlation between mother and baby irisin levels, as expected, was found in this study. There was a strong correlation also between maternal insulin and infant irisin levels in SGA infants, while in LGA infants a strong correlation between infant insulin and leptin levels were found. In SGA infants, elevation of irisin levels can be considered as a protective mechanism for high insulin levels in the mother. In LGA babies, the correlation between leptin and irisin suggests that increased fat tissue increases the effect of both hormones.

When the infants were evaluated from the developmental point of view, it can be concluded that the homeostasis system works well, especially in mothers who gained normal weight and in babies born of normal weight and especially when irisin correlates negatively with all measurements.

The effects of irisin on fetal growth have not yet been elucidated precisely. It is known that obesity, type 2 diabetes and the metabolic syndrome, insulin resistance and consequently increased risks of cardiovascular disease can be programmed in the intrauterine period. Disturbances in intrauterine growth are an important factor in this programming. In this context, further studies are needed to identify and prevent possible predisposing factors to abnormal growth in fetal life and to better understand the mechanism of action of irisin.

## Figures and Tables

**Table 1:**
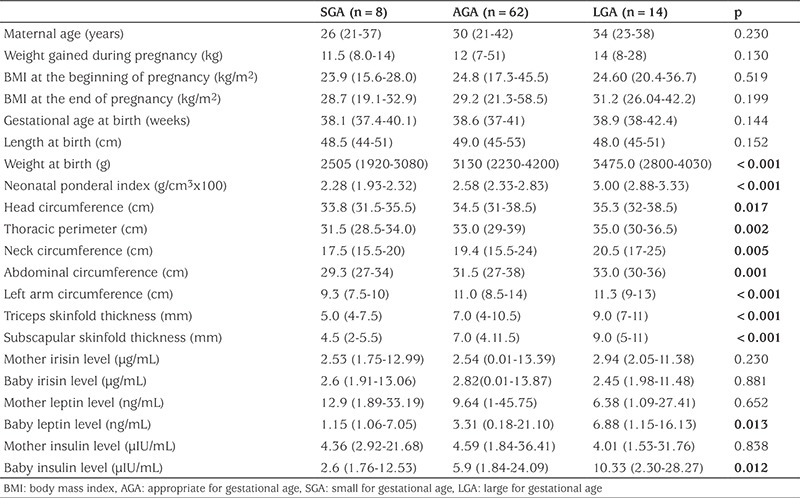
Anthropometric and hormonal parameters in small for gestational age, appropriate for gestational age and large for gestational age infants and their mothers (median values and ranges)

**Table 2:**
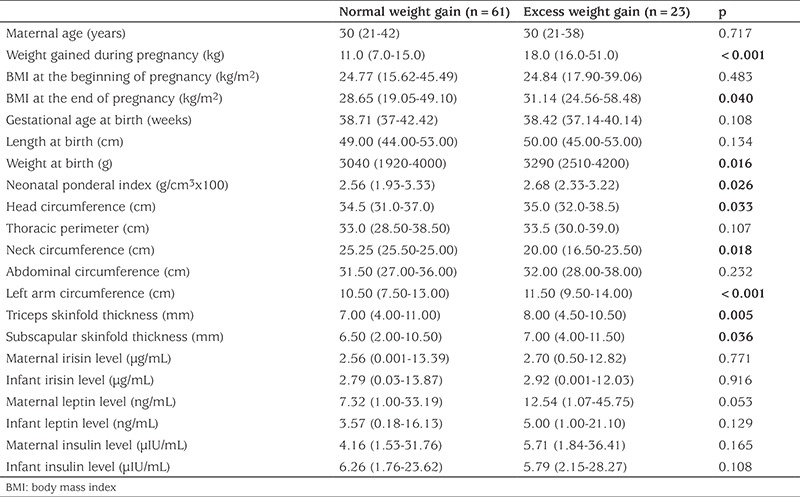
Maternal and infant anthropometric and hormonal parameters according to the weight gain of the mother during pregnancy (median values and ranges)

**Table 3:**
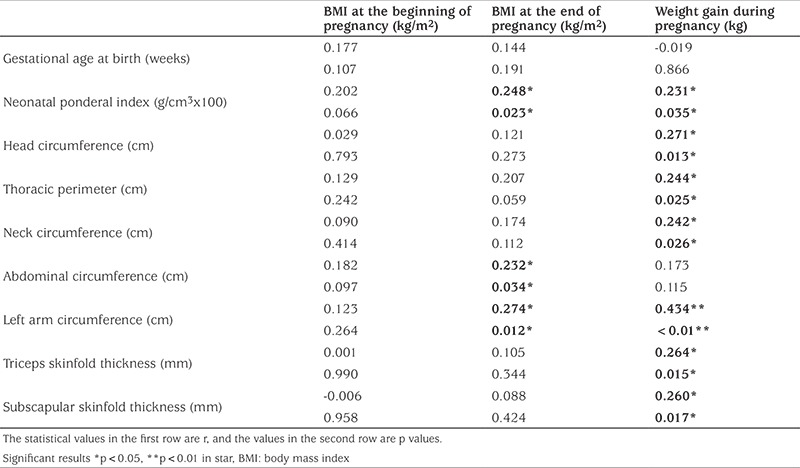
Relationships between maternal weight during pregnancy and anthropometric measurements of the infant

**Table 4:**
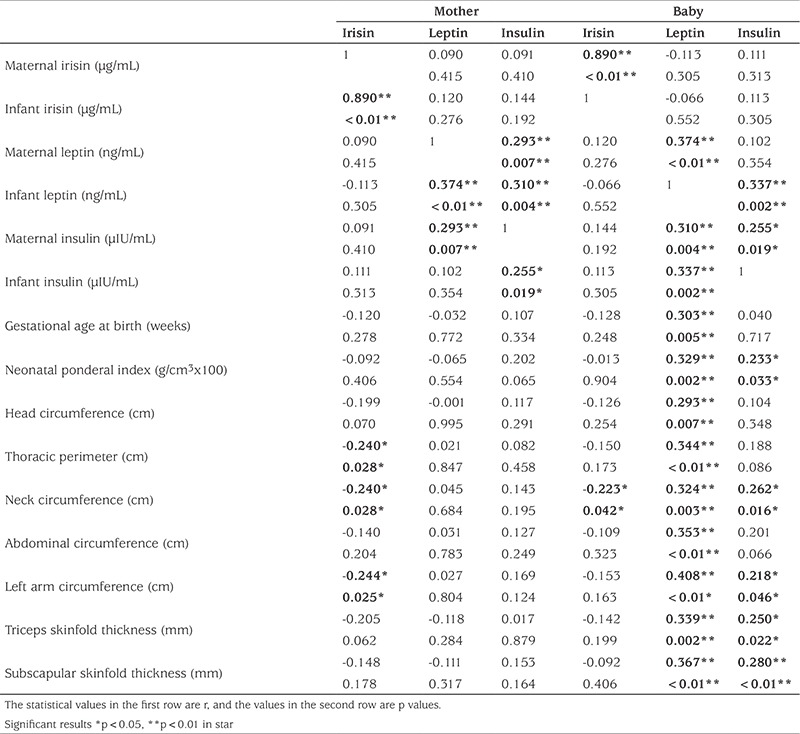
Relationships between maternal and infant hormone levels and anthropometric measurements
